# The Water Bug as a Carrier of Infection

**Published:** 1916-02

**Authors:** 


### The Water Bug as a Carrier of Infection.

Any one who will look up the words water bug, roach, cock roach, croton bug, in Webster, will understand the difficulty in accurately defining the subject of this article and will note an interesting discrepancy between the dictionary definitions and the common local use of the term. However, it is perhaps just as well to ignore questions of entomologic detail and to confine our attention to the more practical points bearing on the possibility of disease conveyance.

The water bug is misnamed. It is easily drowned and is not especially attracted by dampness. It might more properly be called the water-pipe bug but only because pipes afford it avenues of approach, apparently no greater favorites than cracks in wood work and tiling, loose paper, etc. It is not markedly an indication of filth, although accumulations of garbage attract it. But it is equally attracted by fresh foods left exposed, transient accumulations of crumbs, fruit parings, etc., and it is not discouraged by scouring floors, walls, etc. It is, to some degree, nocturnal in its habits but probably merely because less subject to human disturbance than in the day time. One is tempted to ascribe considerable intelligence to the water bug, so rapidly does it seek a place of hiding on the mere approach of man and so agile is it in dodging attempts at capture. It will also evade various bug powders if left in heaps or if in making a ring around a room, the door ways are left free. The quickness with which it senses food suggests a well developed olfactory organ.

In common with practically all insects, it is killed by any powder, even if innocuous to higher animals, on account of mechanic blocking of the breathing tubes. But a safe powder becomes innocuous to the insect also as it becomes moist, as it inevitably does after a few days; powders are objectionable to housewives, and the bug avoids the powder and is only temporarily discouraged from his visits. Curiously enough, the water bug appears to avoid rooms with carpets and rugs and confines himself mostly to kitchens, pantries, halls and bath rooms. At first thought, the explanation appears to be the call of food but this scarcely applies to bath rooms nor do crumbs in dining rooms especially attract the bug. Neither does the explanation as to pipes apply, for radiator pipes in ordinary living rooms afford almost as good entries as those of bath rooms and kitchens, especially in summer when they are not hot.

As in campaigns against other vermin, co-operation is necessary. A separate house can be rid of the pest by persistence but, in hotels and flat buildings, no amount of cleanliness and extermination is of avail except temporarily, unless undertaken for the whole building at once and, even then, fresh hoards sooner or later arrive from some other source.

It is scarcely necessary to undertake bacteriologic experiments to show the reality of the danger of the water bug especially as this danger, in actual environments is limited to non-specific germs and to the accidental presence of specific germs. Recently, it has been claimed to carry an alga that causes cancer—an English theory as yet without demonstration. The water bug crawls rapidly and for considerable distances, without much regard for gravity. It gets onto towels, wash cloths, tooth brushes, etc., in the bath room, on to dishes and food in the pantry and kitchen. It brings whatever germs may be attached in passage along floors, over garbage, over small leaks in closets and drains.

Fly paper catches a few of the smaller and less experienced bugs, bichlorid, potassium oxalate, hydronaphthol and various other antiseptics in safe strength are inefficient. Ordinary methods by powders, low temperature flames, odorous substances such as gasoline, turpentine, etc., and individual slaughter are either of temporary and local benefit, unsatisfactory or otherwise objectionable. A serious problem confronts sanitarians in regard to this little pest.

